# On the Urinary Stones and Calculi in Men

**Published:** 1803-03-01

**Authors:** 


### On the Urinary Stones *and* Calculi *in* Men; *by* Cit. Fourcroy.

[ Concluded from Vol. vii. pp. 60—66. ]

Sect. 5. On *the dissolvents of urinary calculi*. Every thing which has been said or proposed concerning the dissolution of urinary concretions, contained in the bladder of men, and in the urinary passages, must have been inaccurate, till analysis had thrown light on their nature and difference; and though accident has discovered some remedies, adapted to a certain species of calculi, as, the remedy of Mrs. Stephens, the lime water of Mr. Whyte, the caustic lye of Hartley, yet it appears evident, that these celebrated remedies will prove inefficacious in stones originating from terreous phosphats and oxalats of lime, and merely be of service in calculi arising from lithic or uric acid and urats of ammonia. It would be equally superfluous and tedious to prosecute the long series of errors, prejudices and hypotheses, with which the annals of medicine are overspread with respect to the different lithontriptics from the earliest times of the Greek school to the present age. There is hardly a natural substance which we do not find in this extensive catalogue, particularly mineral waters, decoction of plants, sometimes of the most useless; and our researches will confirm the sad observation, so long time since experienced, that the human mind must advance through errors to find the path of truth. In the present age, however, the lithontriptics so much boasted of are disregarded, and their power is discredited, formerly supposed to be fully efficacious, notwithstanding their long passage through the organs of digestion; and it is almost generally acknowledged, that if there is any hope of dissolving calculi, the remedies should be immediately introduced into the bladder through the urethra; on which circumstance the success of Stephens's, Whyte's and Guthrie's remedies seems equally to depend. Repeated experience will probably remove or diminish the fear of danger, that might arise from the sensibility of the parts. Every other way of employing lithontriptics, besides the inconvenience of a long and unmeasurable passage, is attended with so many accidents, capable of diminishing the energy of lithontriptics in the organs they are to pass through, before they arrive at the calculus, that their intended success is mostly frustrated. According to the present state of our knowledge, three or four substances are sufficient for dissolving all species and strata of calculous concretions. The lyes of caustic kali and natron, so much diluted as to be suffered in the mouth and swallowed, are able to soften, liquefy, and to dissolve the lithic acid within a few days, or the small calculi and pieces of the larger ones, if they are put into them. This is likewise the case with urats of ammonia. Nitric and muriatic acid, very much diluted, so as to be not much sharper than urine itself, soften and dissolve still better phosphat of lime and of ammoniacal magnesia; and if these substances are hung by means of a thread in these acids, they become light, swim on the surface, and leave behind transparent flakes, similar to the slimy textures which swim on the surface of the liquor. The presence of uric acid in the lye of potash manifests itself by the addition of a weak acid and even of vinegar, which precipitates it in form of a white powder, and the presence of phosphats by the ammonia, which separate them. Stones consisting of oxalat of lime, and resembling mulberries, are only with difficulty soluble in weak reagents. They are however, softened and almost wholly liquefied in diluted nitric acid, and nothing but a spongy and brownish animal matter remains; but they require a longer time for their solution than the preceding ones. We likewise succeed in dissolving them by means of a lye of carbonat of kali or natron, which by a double affinity decomposes the phosphat of lime; carbonat of lime is thus precipitated from the liquor, in which the oxalat of kali or of natron remains dissolved. It appears therefore, that either of the above mentioned liquid reagents is to act on the calculus and to dissolve it on being injected into the bladder, if no obstacle be opposed to its operation; but here we meet with several difficulties, which it is necessary to know, in order to be able to remove them. The first is, to determine the nature of the calculus, the differences and strata of which may obstruct the successful application of lithontriptics, a circumstance which cannot be avoided but by a proper choice of the remedies, founded on the knowledge of these concretions and their nature. The second difficulty consists in limiting the action of the remedy on the calculus, and in defending the bladder from its attack. The third relates to the mixture of the reagent with the urine, which is capable of modifying, and even of annihilating its effects, and may be attended with inconveniences prejudicial to its operation on the calculus. We shall trace each of these difficulties, and endeavour to prove, that they oppose no insuperable impediments to the solution tion of urinary calculi in men. There are very few expedients, by which we may ascertain from without the nature of urinary concretions, and though the introduction of the catheter is able to inform us of their size, hardness and surface, we can never become acquainted by these means with their composition. No symptom throws light on this subject; and indeed, the difference of the particles composing a calculus, seems to have been utterly disregarded in surgery. The idea of a lithontriptic has never been grounded on the different nature of these concretions, as ought to have been done. Under these circumstances, as the art preserves an absolute silence on this subject, we have thought, that the examination of the urine of calculous persons might furnish us with some ideas on the species of calculi, suspecting that this urine must contain that substance in a less proportion, which it continually accumulates on the exterior of urinary concretions. The examination of the urine from two calculous persons has shown a very perceptible diminution, and almost a total absence of uric acid, which is generally contained in healthy urine; and this circumstance led us to conclude, that their calculi were formed of uric acid. In one, who died of age and infirmity, we actually discovered a stone, consisting of lithic acid. This subject, however, is still so new, that it needs farther enquiries, to confirm or suppress this opinion. The gravel excerned before the symptoms of a calculus appear manifest, may furnish a hint with regard to the nature of this calculus, and our knowledge may be assisted by the calculis, that are shown in families, where they have been evacuated in a natural manner or extracted by an operation, as it is probable that the hereditary disposition towards this disease is owing to one and the same cause, and that consequently the calculous matter, which occurs in one of them, may be likewise one and the same in others of the family. It is to be suggested that amongst all calculous substances, the uric acid and the urats of ammonia are most frequently met with, so as to form at least one-third of all human calculi; while the other two-thirds are constituted by the remaining three substances, viz. the two terreous phosphats and the oxalat of kali; as the siliceous earth, on account of its rarity, deserves hardly to be mentioned; whence it appears, that we have often ground for choosing the lye of kali as a lithontriptic. The continued use of the injections of this remedy does not leave us long undetermined concerning its effect and the nature of the calculus, and in proportion as the symptoms symptoms abate and the size of the calculus decreases, we are confirmed in the nature of the concretion and the choice of the remedy. In the opposite case, however, we ought to have recourse to acids. There is another expedient for ascertaining the nature of urinary calculi and of determining the dissolvent, that is, to examine the remedy a quarter of an hour after its having been injected. On employing a diluted solution, which, however, we know is capable of dissolving concretions of uric acid, we are to collect the liquor after its having remained in the bladder for half an hour or three quarters of an hour, and suffer it to depose the flakes which are mixed with it; and having filtrated it through blossom paper, add some muriatic acid. If the liquor has acted on a calculus of uric acid, and commenced its solution, the muriatic acid will produce a considerable white precipitation. This experiment being continued for several days one after another, and after every injection, will enable us to determine the nature of the acid in the calculus, and even the quantity of the acid which is disengaged from the stone, till the fortunate moment when the disappearance of all symptoms, occasioned by the presence of the calculus, the probe, and the absence of uric acid in the injected liquor, announce and ascertain the total solution of the calculus. Whence it appears evident, that if the alkaline liquor betrays no traces of uric acid, tried in the above manner for several days, and if the symptoms of the calculus do not abate, we have every reason to suppose, that the calculus is not composed of uric acid, and that we must consequently resort to the application of diluted muriatic acid, for the purpose of dissolving this calculus. This acid acts expeditiously on phosphats, and will soon prove efficacious, if the calculus or some of its strata should be composed of them On adding a few drops of a solution of ammonia or kali, a white precipitation of phosphat of lime will be produced, which is separated from the acid by means of the alkali. In this case the symptoms will abate with the diminution of the calculus, which is easily performed by the muriatic acid, in which the phosphats are more soluble than in any other acid. If the stone be formed by two substances, alternately placed one over another, the injected acid will sometimes become no farther efficacious, as is found by the addition of ammonia yielding no precipitation, and then the alkaline lyes should be again employed. The diagnosis of the mulberry-like stones, is attended with still greater difficulties, and we discover no other traces of oxalat of lime in the urine, but its being turbid on passing out of the bladder. By analysis we might expect to find in the precipitation oxalat of lime. However, no such urine has been found and analysed in calculous persons. We do not deny the possibility of discovering it in the urine of calculous persons, issuing from the bladder as a turbid liquor, although a continued examination of the urine of calculous persons will be necessary, undertaken with great care, and through a long series of experiments. In this manner we may perhaps be informed, whether a calculus of oxalat of lime is to be discovered by the analysis of the urine, and whether it may be dissolved by nitric acid and carbonat of kali, as it is done out of the body. Not without much reason it has been apprehended, that the liquors acting on the urinary concretions, might attack the membranes of the bladder and destroy its organization, instead of dissolving the calculi; but such an accident may be easily avoided, as will appear from my experiments. A solution of kali and the acids were so much diluted by water, that they could be suffered in the mouth without any inconvenience, and no greater irritation of the bladder was produced by them than by common urine. I have seen six persons, who had used the alkaline injections, without experiencing any pain or disagreeable sensations arising from the liquor being detained in the bladder. The bladder, however, became more sensible when a weak muriatic acid had been injected, and the irritation thence arising, did not suffer the liquor to remain long in the bladder; but fortunately the muriatic acid, even when extremely diluted, easily dissolves the phosphatic stones, without being for so long a time in contact with them, as is required in the action of alkali on the uric acid. The action, which the lithontriptics and the urine exert upon each other, remains still to be considered. The alkali must meet with free phosphoric and uric acid, by which it is absorbed or saturated, before it can act on the calculus, and in this manner the urine becomes an impediment to its action. There are, however, two expedients to remedy this inconvenience; the first is, to let the urine be evacuated before the injection is made, and to inject previously luke-warm water. The liquor, however, may be weakened by the urine issuing from the ureters; but this is counteracted by the second remedy, or, by letting the patient drink a very diluted solution of kali. The experiments made at Paris and Dijon, shewed, several days after, the use of the pure alkali given internally; the urine became rather alkaline, lost its acidity, and obtained a similar nature to that of the injected liquor.

liquor. It has even been hoped, that we might render the urine so alkaline as to dissolve the stone; and though we cannot conceive that this method will succeed in dissolving great calculi, yet it may be of great service in the gravel of the kidneys, and in hindering the increase of calculi, and in promoting the effect of alkaline injections. There is another inconveniency attending the mixture of urine, and of the injected liquors in the bladder. The former being acid and saturated with the lye, deposes a gelatinous substance in form of slimy flakes, which frequently obstruct the orifices of the catheter, from which they must be removed by means of a wire. The muriatic acid never occasions any of these inconveniences; it renders the urine clearer, and even prevents the deposition of the slimy matter.

The liquors to be injected, ought to have a temperature of 25° Reaum. A catheter of gum elastic and a syringe of tin are the apparatus necessary for this operation. In the beginning it is sufficient to make the injection three or four times a day, afterwards six or eight times, and the liquor is suffered to remain in the bladder for a period not less than a quarter of an hour. The injections must be continued for some time, and the catheter remain in the urethra, that the patient may be enabled to make the injections himself, which he learns to do without difficulty. It is advisable to throw warm water into the bladder after every injection.

